# Gait mechanics in patients with chronic obstructive pulmonary disease

**DOI:** 10.1186/s12931-015-0187-5

**Published:** 2015-02-28

**Authors:** Jennifer M Yentes, Kendra K Schmid, Daniel Blanke, Debra J Romberger, Stephen I Rennard, Nicholas Stergiou

**Affiliations:** Biomechanics Research Building, University of Nebraska at Omaha, 6001 Dodge Street, Omaha, NE 68182-0860 USA; College of Public Health, University of Nebraska Medical Center, 984355 Nebraska Medical Center, Omaha, NE 68198 USA; Nebraska-Western Iowa Veterans’ Health Care System; U.S. Department of Veterans’ Affairs, 4101 Woolworth Avenue, Omaha, NE 68105 USA; Department of Pulmonary and Critical Care Medicine, University of Nebraska Medical Center, 036 DRC2, Omaha, NE 68198-5910 USA

**Keywords:** Pulmonary disease, Locomotion, Joint kinematics, Joint kinetics, Biomechanics

## Abstract

**Background:**

Chronic obstructive pulmonary disease (COPD) is characterized by the frequent association of disease outside the lung. The objective of this study was to determine the presence of biomechanical gait abnormalities in COPD patients compared to healthy controls while well rested and without rest.

**Methods:**

Patients with COPD (N = 17) and aged-matched, healthy controls (N = 21) walked at their self-selected pace down a 10-meter walkway while biomechanical gait variables were collected. A one-minute rest was given between each of the five collected trials to prevent tiredness (REST condition). Patients with COPD then walked at a self-selected pace on a treadmill until the onset of self-reported breathlessness or leg tiredness. Subjects immediately underwent gait analysis with no rest between each of the five collected trials (NO REST condition). Statistical models with and without covariates age, gender, and smoking history were used.

**Results:**

After adjusting for covariates, COPD patients demonstrated more ankle power absorption in mid-stance (*P* = 0.006) than controls during both conditions. Both groups during NO REST demonstrated increased gait speed (*P* = 0.04), stride length (*P* = 0.03), and peak hip flexion (*P* = 0.04) with decreased plantarflexion moment (*P* = 0.04) and increased knee power absorption (*P* = 0.04) as compared to REST. A significant interaction revealed that peak ankle dorsiflexion moment was maintained from REST to NO REST for COPD but increased for controls (*P* < 0.01). Stratifying by disease severity did not alter these findings, except that step width decreased in NO REST as compared to REST (*P* = 0.01). Standardized effect sizes of significant effects varied from 0.5 to 0.98.

**Conclusions:**

Patients with COPD appear to demonstrate biomechanical gait changes at the ankle as compared to healthy controls. This was seen not only in increased peak ankle power absorption during no rest but was also demonstrated by a lack of increase in peak ankle dorsiflexion moment from the REST to the NO REST condition as compared to the healthy controls. Furthermore, a wider step width has been associated with fall risk and this could account for the increased incidence of falls in patients with COPD.

## Background

Chronic obstructive pulmonary disease (COPD) is a worldwide health concern and is one of the few causes of death that is on the rise [1]. COPD affects more than the lungs and Fabbri and Rabe have emphasized the ‘systemic and complex abnormalities’ affecting peripheral systems in patients with COPD [2]. One extra-pulmonary manifestation of COPD is abnormal skeletal muscle structure (i.e., muscular dysfunction). Patients with COPD suffer from abnormal body mass alterations, muscle wasting/atrophy, impaired energy production and metabolic performance, and increased susceptibility to muscle fatigue and weakness [3-11].

Possibly related to abnormal skeletal muscle structure is the decline in ambulatory activity that is common in patients with COPD. Using the 6-minute walk distance test, patients with COPD walked a significantly shorter distance than aged-matched controls [12] and hospitalized patients with COPD walked less than 10 minutes/day during hospitalization and only up to 15 minutes/day after one month of discharge [13]. One third of patients with severe COPD walked less than 15 minutes/day [14] and when compared over a 12-hour time frame, walked 50% less than age-matched controls [15]. Ambulatory activity may be limited in patients with COPD due to neural and/or leg fatigue [16-18], suggesting muscular dysfunction as a major contributor to limitation of ambulation [19].

Gait abnormalities may be present in COPD that are distinct from muscular weakness. Specifically, abnormalities in the biomechanics of the gait cycle may contribute to altered ambulation. Our previous examination into existing data consisting of subjective reports of gait abnormalities in patients with COPD, demonstrated that the presence of COPD is associated with the report of a limp, shuffle or other walking abnormality, which appeared to be related to COPD severity [20]. However, objective analysis of gait mechanics in patients with COPD has yet to be completed. Annegarn and colleagues used an accelerometer to assess gait during a 6-minute walk test and demonstrated that cadence, walk intensity and variability were related to the distance walked in COPD patients [21]. More detailed analysis of lower extremity joint muscular responses can be provided using advanced biomechanical analysis in the form of joint angles, moments, and powers [22-24]. Joint moments can determine the net response of all muscle groups in the lower extremities revealing crucial COPD-related adaptations. Whereas, joint powers determine the net contribution of all muscle groups at that joint. This analysis has been used extensively to understand the control mechanisms in healthy individuals and in gait abnormalities of patients with cerebral palsy [25-27], stroke [28-30], head injury [31-33] and other neuromuscular disorders [34-36].

Hence, the overall objectives of this study were to objectively and comprehensively characterize gait abnormalities present in patients with COPD. Utilizing the mechanical analysis of joint angles, moments, and powers, in addition to spatiotemporal gait parameters, in persons with COPD and aged-matched controls, the presence of abnormal gait patterns was assessed. Based upon our previous work [20], it was hypothesized that patients with COPD would demonstrate decrements in their gait as compared to healthy controls. Gait analysis was performed in two conditions, REST and NO REST. The NO REST condition was provided to the subjects to investigate an activity-induced condition. Specifically, it was hypothesized that a lack of rest would exacerbate alterations in gait mechanics. Furthermore, our previous study indicated that disease severity, age, gender, and smoking history may be associated with gait abnormalities in patients with COPD. Thus, statistical models were used to limit the effects of these potential confounding variables.

## Methods and materials

Patients with COPD were recruited from the Pulmonary Clinical Studies Unit and general clinics of local hospitals. A FEV_1/_FVC ratio of 0.7 was used to define the presence of COPD [37]. Participants were excluded if there was a history of back or lower extremity injury or surgery that affected the subject’s mobility or any other process limiting the ability to walk, including neurological disease or impairment. In total, 17 patients with COPD and 21 age-, height- and weight-matched healthy controls were consented and participated in this study (Table 1). The University Institutional Review Board and the Institutional Review Board at the Omaha Veterans’ Affairs Medical Center approved all procedures and written informed consent was obtained from all patients.

**Table 1:** **Subject characteristics**

	**Control mean (SD) n = 21**		**COPD mean (SD) n = 17**	***P***
**Gender (n)**	Male = 10		Male = 11	
**Age (years)**	65.33 (7.67)		63.77 (8.55)	0.56
**Height (cm)**	165.89 (16.43)		171.97 (11.79)	0.21
**Weight (kg)**	78.85 (18.08)		90.58 (25.62)	0.11
**FEV**_**1**_ **/FVC**	0.74 (0.04)		0.51 (0.16)	<0.001*
**FEV**_**1**_ **% Predicted**	101.71 (11.00)		50.18 (21.0)	<0.001*
**Smoking History (n)**				
Current Smoker	2		5	
Ex-Smoker	7		11	
Never Smoker	12		1	
	Control Rest Mean (SD)	Control No Rest Mean (SD)	COPD Rest Mean (SD)	COPD No Rest Mean (SD)
**Speed (m/s)**	1.09 (0.16)	1.07 (0.25)	1.11 (0.17)	1.15 (0.18)
**Step Length (m)**	0.66 (0.07)	0.65 (0.09)	0.66 (0.06)	0.66 (0.06)
**Step Width (m)**	0.11 (0.03)	0.11 (0.03)	0.12 (0.04)	0.11 (0.04)
**Step Time (sec)**	0.60 (0.06)	0.62 (0.06)	0.59 (0.06)	0.58 (0.06)
**Stance Time (sec)**	0.70 (0.09)	0.73 (0.08)	0.70 (0.10)	0.69 (0.09)
**Support Time (sec)**	0.11 (0.03)	0.12 (0.02)	0.12 (0.04)	0.11 (0.03)
**Stride Length (m)**	1.32 (0.14)	1.33 (0.17)	1.31 (0.13)	1.33 (0.13)
**Stride Time (seconds)**	1.19 (0.13)	1.22 (0.12)	1.18 (0.13)	1.15 (0.11)

Note: * indicates significance *P* < 0.05.

### Protocol

To assess gait, reflective markers were placed on defined anatomical locations, bilaterally, according to a modified Helen Hayes marker set [38]. Participants were asked to walk through a 10-meter walkway at a normal pace. To ensure that a complete footfall would be collected during each trial, starting positions for each limb were determined prior to data collection. Five trials were collected for each limb, 10 trials total. The 3D marker trajectories were collected with a high-speed motion capture system (Motion Analysis Corp., Santa Rosa, CA; 60 Hz). Ground reaction force data from heel contact to toe off were collected using a piezoelectric force plate (Kistler Instrument Corp., Winterthur, Switzerland; 600 Hz). All participants were given a minimum one-minute rest between each trial during the data collection (REST condition).

All patients with COPD were then asked to determine their self-selected pace on a treadmill at 0% incline. They then walked at their chosen self-selected pace at 10% incline until the onset of self-reported breathlessness or muscle tiredness (reported as either the development of shortness of breath or the onset of subjective muscular fatigue). Subjects were asked to walk until they felt like they could not continue any further due to breathlessness or muscle tiredness. This was a completely subjective interpretation. Motion capture data was not collected while the subject walked on the treadmill. Once self-reported breathlessness or muscle tiredness was reported, they were immediately removed from the treadmill and asked to walk through the 10-meter walkway, 5 times for each limb, with no rest in between trials (NO REST condition). A subset (n = 5) of healthy controls also went through the NO REST condition in order to investigate the main effect of condition in non-COPD group. Since these subjects were not expected to indicate tiredness or breathlessness, they were asked to walk for 15 minutes at a self-selected speed on the treadmill at a 10% grade.

### Data analysis

Gait variables were calculated from the five trials during the stance phase of walking for each subject (Table 2). Each marker’s three directions were filtered using the Jackson algorithm [39] with cutoff values ranging from 2-8Hz. Visual 3D (C-Motion, Inc., Germantown, Maryland) was used for calculation of joint angles, moments, and powers. A standing calibration was used to align the local reference frames of the segments to the global reference frame. A hybrid model was built using anthropometric data from Dempster [40]. Custom programs (MatLab 2007, Mathworks, Inc., Concord, MA) were used to pick peak angles, moments, and powers from calculated joint curves. In the REST condition, two healthy controls and one patient with COPD did not have a sufficient number of strides collected for spatiotemporal data analysis. One healthy control and one patient with COPD did not have a sufficient number of strides collected for spatiotemporal data analysis in the NO REST condition. They were removed for spatiotemporal analysis even though they were included in the kinematic and kinetic analysis. One patient with COPD could not complete the NO REST condition due to exhaustion.

**Table 2:** **Dependent variables and their descriptions**

**Dependent variable**	**Description**
**Speed (m/s)**	Measured as the derivative of the position of the sacral marker.
**Step Length (m)**	Anterior-posterior distance from the heel strike of the right foot to the heel strike of the left foot.
**Step Width (m)**	Medial-lateral distance from the heel strike of the right foot to the heel strike of the left foot.
**Step Time (seconds)**	Time from the heel strike of the right foot to the heel strike of the left foot.
**Stance Time (seconds)**	Time between heel strike and toe off for the right foot.
**Double Support Time (seconds)**	Timing of the heel strike of the left foot to the toe off of the right foot (terminal double support).
**Stride Length (m)**	Anterior-posterior distance from two consecutive right heel strikes.
**Stride Time (seconds)**	Time between two consecutive right heel strikes.
**Peak Plantarflexion Angle (deg)**	Minimum angle during early stance.
**Peak Dorsiflexion Angle (deg)**	Maximum positive angle during late stance.
**Peak Knee Flexion Angle (deg)**	Maximum positive angle during early to mid stance.
**Peak Knee Extension Angle (deg)**	Minimum angle (close to zero) during mid to late stance.
**Peak Hip Flexion Angle (deg)**	Maximum positive angle at very early stance.
**Peak Hip Extension Angle (deg)**	Minimum angle (close to zero) during late stance.
Peak Dorsiflexion Moment (N*m/kg)	Minimum rotational force during early stance.
Peak Plantarflexion Moment (N*m/kg)	Maximum rotational force during late stance.
Peak Knee Extension Moment (N*m/kg)	Maximum rotational force during mid stance.
Peak Knee Flexion Moment (N*m/kg)	Minimum rotational force during mid to late stance.
Peak Hip Extension Moment (N*m/kg)	Maximum rotational force during very early stance.
Peak Hip Flexion Moment (N*m/kg)	Minimum rotational force during late stance.
**Peak Ankle Power Absorption 1 (W/kg)**	Minimum energy absorbed during early stance.
**Peak Ankle Power Absorption 2 (W/kg)**	Minimum energy absorbed during mid to late stance.
**Peak Ankle Power Generation (W/kg)**	Maximum energy generated during late stance.
**Peak Knee Power Absorption 1 (W/kg)**	Minimum energy absorbed during early to mid stance.
**Peak Knee Power Generation (W/kg)**	Maximum energy generated during mid stance.
**Peak Knee Power Absorption 2 (W/kg)**	Minimum energy absorbed during late stance.
**Peak Hip Power Generation 1 (W/kg)**	Maximum energy generated during early stance.
**Peak Hip Power Absorption (W/kg)**	Minimum energy absorbed during mid to late stance.
**Peak Hip Power Generation 2 (W/kg)**	Maximum energy generated during late stance.

All variables were checked for normality and group means of each dependent variable (Table 2) were calculated for each group (control and COPD) and for each condition (REST and NO REST). Four linear models were utilized on each dependent variable in Table 2:

1. A 2x2 linear model with repeated measures for condition was used to compare outcomes between group (all COPD vs control) and condition (REST vs NO REST) and to assess the group by condition interaction.
2. The covariates of age, gender and smoking history were added to model #1.
3. A 3x2 linear model with repeated measures for condition was used to compare outcomes between group (mild/moderate COPD vs severe/very severe COPD vs control) and condition (REST vs NO REST) and to assess the group by condition interaction. Disease severity was defined using GOLD standards [37]. Tukey’s method was used to adjust for multiple comparisons.
4. The covariates of age, gender and smoking history were added to model #3.

All statistical analysis was done using SAS version 9.3 (SAS Institute Inc., Cary, NC, USA). To control for the unbalanced group sizes, the LS Means were calculated and compared using SAS PROC MIXED. The significance level was set at *P* < 0.05. Standardized effect sizes (ES) were estimated as difference in LSMeans divided by the standard deviation of the difference for all significant findings.

## Results

**Figure 1:**
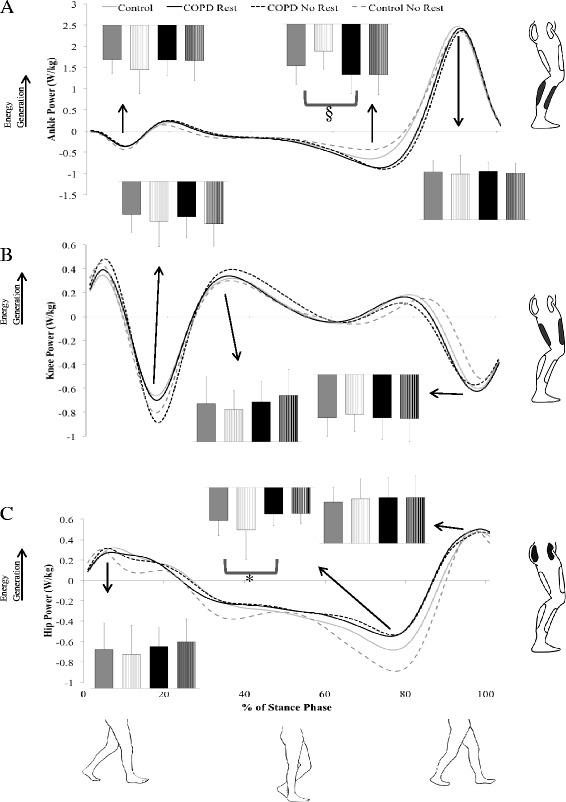
**Sagittal plane joint power mean ensemble curves for the stance phase of gait for the: A) ankle, B) knee, and C) hip.** Positive values represent energy generation. Peak joint powers are shown in the bar graphs for the healthy controls rest (solid gray), healthy controls no rest (striped gray), COPD rest (solid black), and COPD no rest (striped black). Note: * indicates significance (*P* < 0.05) at the indicated peak hip power absorption at mid stance between the healthy controls and patients with COPD. This finding was significant when covariates were not added to the model. Patients with COPD demonstrated less hip power absorption as compared to controls. § indicates significance (*P* < 0.05) at the indicated peak ankle power absorption at mid stance between healthy controls and patients with COPD when covariates were added to the model.

**Figure 2:**
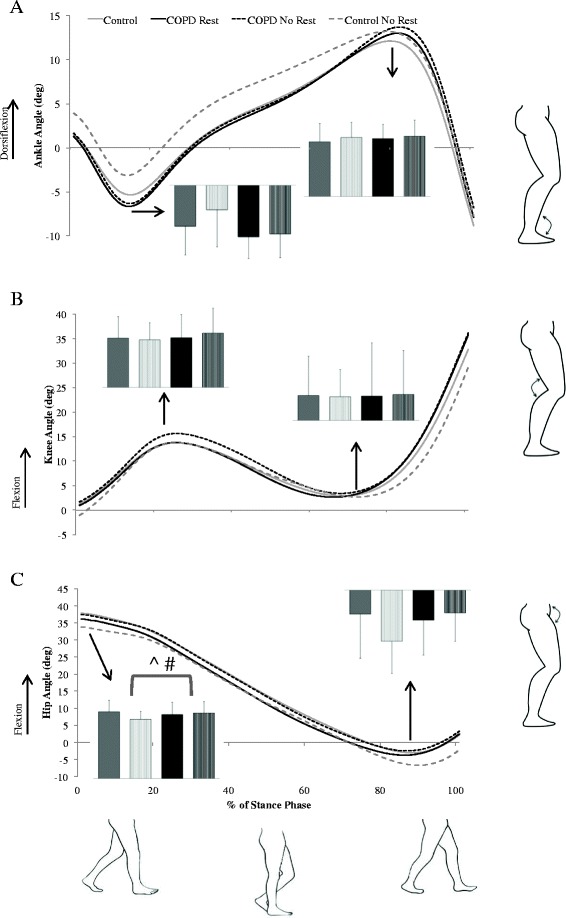
**Sagittal plane joint angle mean ensemble curves for the stance phase of gait for the: A) ankle, B) knee, and C) hip.** Positive values represent dorsiflexion and flexion. Peak joint angles are shown in the bar graphs for the healthy controls rest (solid gray), healthy controls no rest (striped gray), COPD rest (solid black), and COPD no rest (striped black). Note: ^ indicates a significant (*P* < 0.05) main effect of condition (rest vs no rest) in the model without covariates whereas, # indicates the same main effect but in the model in which the covariates age, gender and smoking history have been added. In both models, peak hip flexion angle is increased in the no rest condition.

**Figure 3:**
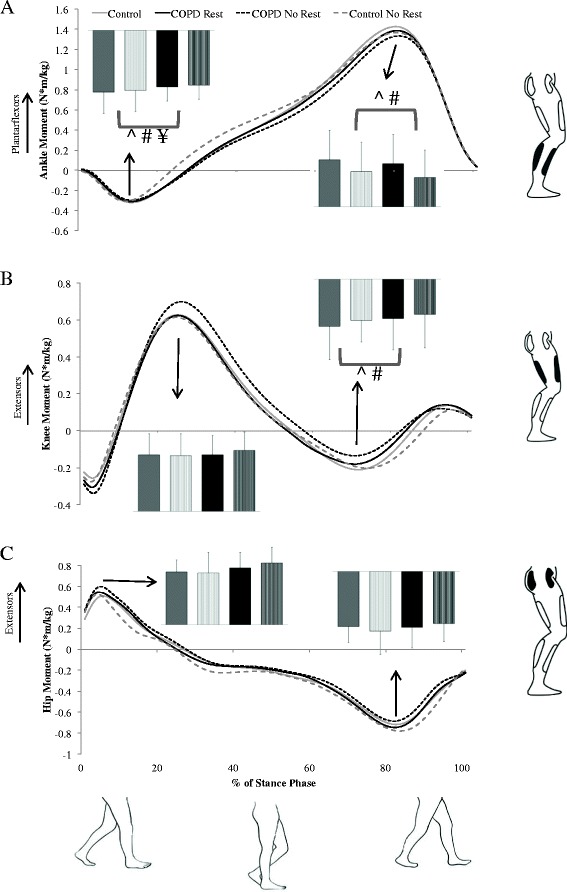
**Sagittal plane joint moment mean ensemble curves for the stance phase of gait for the: A) ankle, B) knee, and C) hip.** Positive values represent plantarflexion and extension. Peak joint moments are shown in the bar graphs for the healthy controls rest (solid gray), healthy controls no rest (striped gray), COPD rest (solid black), and COPD no rest (striped black). Note: ^ indicates a significant (*P* < 0.05) main effect of condition (rest vs no rest) in the model without covariates whereas, # indicates the same main effect but in the model in which the covariates age, gender and smoking history have been added. In both models, peak knee flexion moment is increased and peak ankle plantarflexion moment is decreased in the no rest condition. ¥ indicates a significant (*P* < 0.05) interaction.

**Figure 4:**
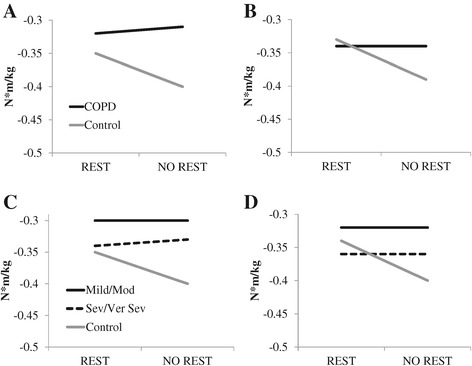
**Significant interaction for peak ankle dorsiflexion moment in early stance found in model #1 (A), model #2 (B), model #3 (C), model #4 (D).** In A and B, patients with COPD remain relatively stable and healthy controls change from REST to NO REST condition, increasing (becoming more negative) their peak ankle dorsiflexion moment. In C and D, patients with COPD have been stratified for disease severity and again, there is a similar pattern. Both mild/moderate and severe/very severe COPD patients maintain a similar peak ankle dorsiflexion moment from REST to NO REST conditions whereas, the healthy controls increase (become more negative) in NO REST.

For model #1, a main effect of group was found for peak hip power absorption at mid-stance (F_1, 36_ = 4.71, *P* = 0.04, ES = 0.7) in which patients with COPD absorb less power than do controls. Several significant main effects of condition were also found. In the REST condition, peak knee power absorption during early stance (F_1, 19_ = 4.70, *P* = 0.04, ES = 0.5; Figure 1) was increased and peak hip flexion angle (F_1, 19_ = 4.71, *P* =0.04, ES = 0.5; Figure 2), peak knee flexion moment (F_1, 19_ = 5.07, *P* = 0.04, ES = 0.5; Figure 3) and peak plantarflexion moment (F_1, 19_ = 5.12, *P* = 0.04, ES = 0.5; Figure 3) were significantly decreased as compared to the NO REST condition. In addition, gait speed and stride length both significantly increased in the NO REST condition as compared to the REST condition (F_1, 17_ = 4.76, *P* = 0.04, ES = 0.5 & F_1, 17_ = 5.86, *P* = 0.03, ES = 0.6 respectively). Lastly, a significant interaction was found for peak ankle dorsiflexion moment in early stance (F_1, 19_ = 16.57, *P* = 0.007; Figure 4A). Patients with COPD slightly decreased their dorsiflexion moment from REST to NO REST (Mean ± standard error: −0.32 ± 0.32 to −0.31 ± 0.02 Nm/kg), whereas healthy controls increased their dorsiflexion moment from REST to NO REST (−0.35 ± 0.02 to −0.40 ± 0.02 Nm/kg).

When covariates were added (model #2), the hip no longer demonstrated a group effect and instead a main effect for group was found at the ankle. Peak ankle power absorption during mid-stance was significantly increased (F_1, 32_ = 8.80, *P* = 0.006, ES = 0.98) in patients with COPD as compared to controls. Significant main effects of condition found similar to those found in model #1, with the exception of the peak knee power absorption in early stance. Peak plantarflexion moment (F_1, 19_ = 5.99, *P* = 0.02, ES = 0.53) was decreased and peak hip flexion angle (F_1, 19_ = 4.98, *P* =0.04, ES = 0.5) and peak knee flexion moment (F_1, 19_ = 5.22, *P* = 0.03, ES = 0.5) were significantly increased in NO REST as compared to REST. Gait speed and stride length both significantly increased in the NO REST condition as compared to the REST condition (F_1, 17_ = 4.80, *P* = 0.04, ES = 0.5 & F_1, 17_ = 5.80, *P* = 0.03, ES = 0.6, respectively). The interaction for peak ankle dorsiflexion moment in early stance maintained significance when covariates were added to the model as well (F_1, 19_ = 17.10, *P* = 0.0006) (Figure 4B). Patients with COPD did not change their dorsiflexion moment from REST to NO REST (Mean ± standard error: −0.34 ± 0.03 Nm/kg for both) but the healthy controls increased their dorsiflexion moment across conditions (−0.33 ± 0.02 to −0.39 ± 0.03 Nm/kg).

In model #3, covariates were not used and patients with COPD were stratified into disease severity classifications: mild/moderate and severe/very severe and compared to controls. No main effect for group was found. Main effects for condition were found for similar variables as before. Peak plantarflexion moment (F_1, 18_ = 8.01, *P* = 0.01, ES = 0.6) was decreased and peak hip flexion angle (F_1, 18_ = 5.59, *P* =0.03, ES = 0.5) and peak knee power absorption during early stance (F_1, 18_ = 5.57, *P* = 0.03, ES = 0.5) were significantly increased in NO REST as compared to REST. Gait speed (F_1, 16_ = 6.72, *P* = 0.02, ES = 0.6) and stride length (F_1, 16_ = 7.55, *P* = 0.01, ES = 0.6) significantly increased in NO REST as compared to REST. Step width (F_1, 16_ = 7.44, *P* = 0.01, ES = 0.6) significantly decreased in NO REST as compared to REST. A significant interaction for peak ankle dorsiflexion moment (F_2, 18_ = 8.19, *P* = 0.003) was found (Figure 4C). Patients with mild to moderate COPD did not change their dorsiflexion moment (Mean ± standard error: −0.30 ± 0.03 Nm/kg for both) across conditions and patients with severe to very severe COPD slightly decreased their moment (−0.34 ± 0.04 Nm/kg to −0.33 ± 0.04 Nm/kg) from REST to NO REST. Healthy controls increased their dorsiflexion moment significantly from −0.35 ± 0.02 Nm/kg to −0.40 ± 0.03 Nm/kg from REST to NO REST.

The last model, #4, stratified patients with COPD into disease severity and utilized the covariates of age, gender, and smoking history. A main effect of group was found for peak ankle power absorption during mid-stance. Patients with COPD had significantly increased (F_2, 31_ = 4.34, *P* = 0.02) power absorption at the ankle in mid-stance as compared to controls. Follow-up comparisons reveal that severe/very severe COPD are increased as compared to controls (t_31_ = 2.54, *P* = 0.04, ES = 0.8). No significant differences were found between mild/moderate compared to controls or mild/moderate compared to severe/very severe. Significant main effects of condition found were identical to those found in model #3. Peak plantarflexion moment (F_1, 18_ = 8.91, *P* = 0.008, ES = 0.65) was decreased and peak hip flexion angle (F_1, 18_ = 5.76, *P* =0.03, ES = 0.5) and peak knee power absorption during early stance (F_1, 18_ = 5.50, *P* = 0.03, ES = 0.5) were significantly increased in NO REST as compared to REST. The same spatiotemporal variables also demonstrated a main effect of condition. Gait speed (F_1, 16_ = 6.80, *P* = 0.02, ES = 0.6) and stride length (F_1, 16_ = 7.51, *P* = 0.01, ES = 0.6) significantly increased in NO REST as compared to REST. Step width (F_1, 16_ = 7.44, *P* = 0.01, ES = 0.6) significantly decreased in NO REST as compared to REST. A significant interaction for peak ankle dorsiflexion moment (F_2, 18_ = 8.43, *P* = 0.003) was found (Figure 4D). Patients with mild to moderate COPD (Mean ± standard error: −0.32 ± 0.04 Nm/kg for both conditions) and severe to very severe COPD (−0.36 ± 0.04 Nm/kg for both conditions) did not change their dorsiflexion moment across conditions. Healthy controls increased their dorsiflexion moment significantly from −0.34 ± 0.03 Nm/kg to −0.40 ± 0.03 Nm/kg from REST to NO REST.

## Discussion

The purpose of this experiment was to characterize biomechanical gait abnormalities in patients with COPD as compared to their healthy counterparts. Contrary to our hypothesis, patients with COPD demonstrated minimal significant differences in biomechanical gait analysis when walking under a REST condition (only one main effect of group was found for all dependent variables in models #1, #2, and #4). Patients with COPD do demonstrate biomechanical gait changes at the ankle as compared to healthy controls. This was seen not only in peak ankle power absorption during NO REST but also patients with COPD demonstrate a lack of increase in peak ankle dorsiflexion moment from the REST to the NO REST condition as compared to the healthy controls. We had hypothesized that they would indeed suffer from gait decrements based on our previous findings [20]. During our previous study, we had investigated the presence of gait abnormalities reported in the publicly available NHANES III dataset and the gait abnormalities reported were subjective and based on observation. The lack of significant findings in the current study may be related to the inherent heterogeneity in which COPD presents itself clinically. Recently, several potential phenotypes (subset) of the COPD syndrome have been identified including a clinical phenotype (age, gender, smoking history), physiological (rapid decline in FEV_1_), radiographic or imaging (structural abnormalities), acute exacerbation of COPD, systemic inflammation, and the presence of co-morbidities (cardiovascular disease, metabolic syndrome, osteoporosis, diabetes, depression) [41,42]. Thus, it cannot yet be concluded that there is no difference between the gait of healthy controls and patients with COPD in a REST condition, except at the ankle, and such differences may be a feature of specific subset (s) of COPD patients. Further work is needed to clearly define these differences.

The secondary aim was to examine the effect of NO REST on the biomechanical gait parameters of patients with COPD. Based on previous reports of increased presence of muscular fatigue in patients with COPD [4,43], we had hypothesized that their gait would significantly decrement under a NO REST condition as inducing activity may lead to fatigue. Several parameters statistically changed from the REST to NO REST condition for both groups. In the NO REST condition, speed and stride length increased potentially explaining the increased hip flexion angle and knee power absorption at early stance during the NO REST condition. Thus, as the subjects walked faster and took longer steps, their hip would flex more and the knee would absorb more of the energy generated from the increased walking speed. It should be noted that even though both groups walked faster under the NO REST condition, the mean increases in gait speed and stride lengths were quite small, although statistically significant. In addition, the peak ankle plantarflexion moment at push-off decreased for both groups during the NO REST condition.

Interestingly, a significant interaction was found for peak ankle dorsiflexion moment at early stance no matter the statistical model employed. Patients with COPD maintained the same amount of torque in their dorsiflexors from REST to NO REST whereas the healthy controls increased (more negative) their peak dorsiflexion torque in the NO REST condition. For the healthy controls, this makes sense as speed has increased in the NO REST condition, an increased dorsiflexion moment would be needed to control the lowering of the forefoot to the ground. Based on these findings, it appears that the patients with COPD may have problems in controlling the foot shortly after heel strike. Additionally, the longer stride lengths noted in the NO REST condition may place the tibialis anterior under greater mechanical demand and this along with possible muscular fatigue could account for the lack of change in patients with COPD. Muscular fatigue during various physical activities has been reported in patients with COPD [4,44] and up to 40% of patients present with muscular fatigue as their main barrier to physical activity [16]. From the current study, we cannot determine if our findings are truly due to muscular fatigue. The NO REST condition was an activity-induced condition and was not a true fatigue-induced condition and may have been more of a mild exercise or warm-up type condition. Yet, muscular fatigue could have been present although this was not investigated in this study. Future work could investigate fatigue through more demanding protocols or through the use of instrumented treadmills, in which steps from the beginning of a trial are compared to steps at the end of a trial when fatigue is reported.

Overall, the models were very similar in findings. Only slight differences were found with the introduction of the covariates of age, gender, and smoking history. In model #1, the hip was found to be significantly different yet upon adding covariates (model #2), the hip was no longer significant but the ankle was found to be significant. In model #1, the ankle was almost significant, *P* = 0.07. When the covariates were added, there was a significant age effect (*P* = 0.003), so after adjusting for age, there was no difference between groups for the hip, that difference was confounded with age. With the ankle, it was marginally significant in model #1, and became even more pronounced after adjusting for the effect of age, model #2. A similar finding was found with models #3 and #4. A group effect for the ankle was found in model #4 that was not seen in model #3. Without the covariates (model #3), the ankle was marginally significant *P* = 0.11, and after adjusting for the effect of age, it became more pronounced and reached significance. Based upon these findings, age was the only covariate that had an impact on the overall findings. This is expected as aging does have an effect on gait [45]. Gender and smoking history did not confound any of the findings. Gender differences in biomechanical gait studies are typically not reported; however, a few studies have noted differences between genders [46,47]. It is interesting to note that smoking did not affect the findings, as smoking is associated with increased systemic inflammation, which is thought to be a possible mechanism of change in the structure and function of skeletal muscle in patients with COPD [48,49].

In COPD, decreased mitochondrial density and fractional area in the vastus lateralis with decreased oxidative enzymes leads to decreased oxidative capacity [10,50]. Further, oxidative damage, possibly leading to atrophy and muscle wasting [51] has been documented, along with muscle fiber type shifting, where oxidative fiber type (type-I) shifted to glycolytic fiber types (type-II) [4,6,52]. In contrast, it has been shown that the more distal tibialis anterior has normal fractional area and oxidative capacity despite decreased mitochondrial density [50]. Yet, two recent articles demonstrate that muscle weakness is present in the ankle dorsiflexors and plantarflexors, greater than weakness present in the quadriceps of patients with COPD [53,54] and that this weakness is related to changes in muscle structure [54]. Multiple factors could be at play affecting muscle dysfunction and structural changes in COPD [55]. The two most popular schools of thought are 1) disuse and 2) chronic systemic inflammation. However, no conclusive research has yet to be done and appears that possibly a mix of the two mechanisms may be at play and/or two phenotypes exist within the disease. In addition, it is feasible that blood flow and possible changes in blood gasses that are not generally regarded as clinically important could also play a role [56]. Although, for patients with COPD, peripheral muscle oxygenation is not compromised during submaximal exercise [57], rather exercise capacity is limited due to disuse and poor lung function (breathlessness).

A curious and interesting finding during the NO REST condition is that of the changes in step width. In models #3 and #4 when disease severity was entered into the statistical models, it was shown that step width decreased significantly in NO REST as compared to REST. The step width of patients with COPD was narrower in the NO REST condition as compared to their REST condition. Patients with mild to moderate COPD walked with a wider step width under both conditions as compared to controls and patients with severe to very severe COPD. Walking with a wider step could be done as an attempt to increase the base of support during the double support phase of gait. As reported in the introduction, fall risk is increased in patients with COPD [58-61] and changes in step width have been found to be associated with fall risk [62-65]. It appears though that step width variability is the dependent variable associated with fall risk rather than just a mean change in step width [66-68]. Future studies should examine step width variability in patients with COPD and the association of this variability with fall risk and fear of falling.

A limitation of the current study is the heterogeneity of the manifestations of COPD [69-71]. While COPD is defined in terms of fixed airflow limitation, COPD is also characterized by the frequent but extremely variable association of disease outside the lungs [72,73]. Among these, muscle fatigue is a common symptom associated with mild to moderate COPD patients irrespective of lung function, anthropometric data or quadriceps strength [74]. Contributing factors to leg fatigue could be related to abnormal structure and function of muscle tissue in COPD [4,6,9,10]. A gauge of exhaustion or fatigue was not utilized in this study and should be considered in future work. Moreover, several other co-morbidities have been associated with COPD such as osteoporosis [75-77], diabetes [78,79], as well as anxiety and depression [12,80-82], which also associated with gait abnormalities. This does not include the other phenotypes of the disease that have been proposed [41,42]. Likewise, it is possible that our healthy controls did indeed have an associated co-morbidity that would likely result in altered gait mechanics as well. Although all patients were screened by a nurse practitioner before inclusion into the study, it is possible that not all co-morbidities were included in our exclusion criteria. Future studies should focus on the inclusion of just patients with severe disease or increase the sample size for each disease severity.

## Conclusions

The novel finding of this study was that patients with COPD do indeed demonstrate biomechanical gait alterations when walking under a NO REST or activity-induced condition. These documented changes were loss of function at the ankle joint, in that peak ankle dorsiflexion moment did not increase under the NO REST condition as it did in the control sample. Although fatigue was not directly measured in the current study, it is feasible that the patients with COPD did present with leg fatigue when activity was induced. Biomechanical gait analysis can provide insight into the joint muscular responses and contributions to movement, providing insight into mechanisms of functional limitations in these patients. Moreover, considering the heterogeneity of COPD, it is possible that a subset of COPD patients, too small to have meaningful inclusion in the current study, may manifest differences not observed in the larger group. Future work should focus on investigating the effects of muscular fatigue and associations with biomechanical gait decrements in this population. In addition, future work should examine biomechanical abnormalities in the different phenotypes associated with the diagnosis of COPD as some subsets of patients may present with gait abnormalities and some patients may not. Lastly, the finding of widened step width in patients with COPD in the REST condition is of particular interest. The increased incidence of falls in patients with COPD has been reported and step width has been indicated as a factor in fall risk. Further investigations into the association of step width variability and fear of falling and fall risk in patients with COPD could reveal significant findings as well as lead to innovative rehabilitation techniques.

## Abbreviations

COPD: Chronic obstructive pulmonary disease
FEV_1_: Forced expiratory volume in one second
FVC: Forced vital capacity
ES: Effect size
